# Supplementary material for: The structures of protein kinase A in complex with CFTR: Mechanisms of phosphorylation and noncatalytic activation
Source: Proc Natl Acad Sci U S A. 2024 Nov 4;121(46):e2409049121. doi: 10.1073/pnas.2409049121 (PMC11573500; doi:10.1073/pnas.2409049121)
Supplement: Supplementary file 1 — Appendix 01 (PDF) [file pnas.2409049121.sapp.pdf]

Supplementary Information for

The structures of protein kinase A in complex with CFTR: mechanisms of phosphorylation and non-catalytic activation

Karol Fiedorczuk[1,2], Iordan Iordanov[3,4], Csaba Mihályi[3,4], András Szöllősi[3,4], László Csanády[3,4*], and Jue Chen[1,5**]

[1]Laboratory of Membrane Biology and Biophysics, The Rockefeller University, New York, NY 10065, USA.

[2]Current address:

[3]Department of Biochemistry and [4]HUN-REN-SE Ion Channel Research Group, Semmelweis University, Tűzoltó u. 37-47, Budapest, H-1094, Hungary

[5]Howard Hughes Medical Institute, Chevy Chase, MD 20815, USA.

[*]Correspondence: csanady.laszlo@semmelweis.hu

[**]Correspondence: juechen@rockefeller.edu

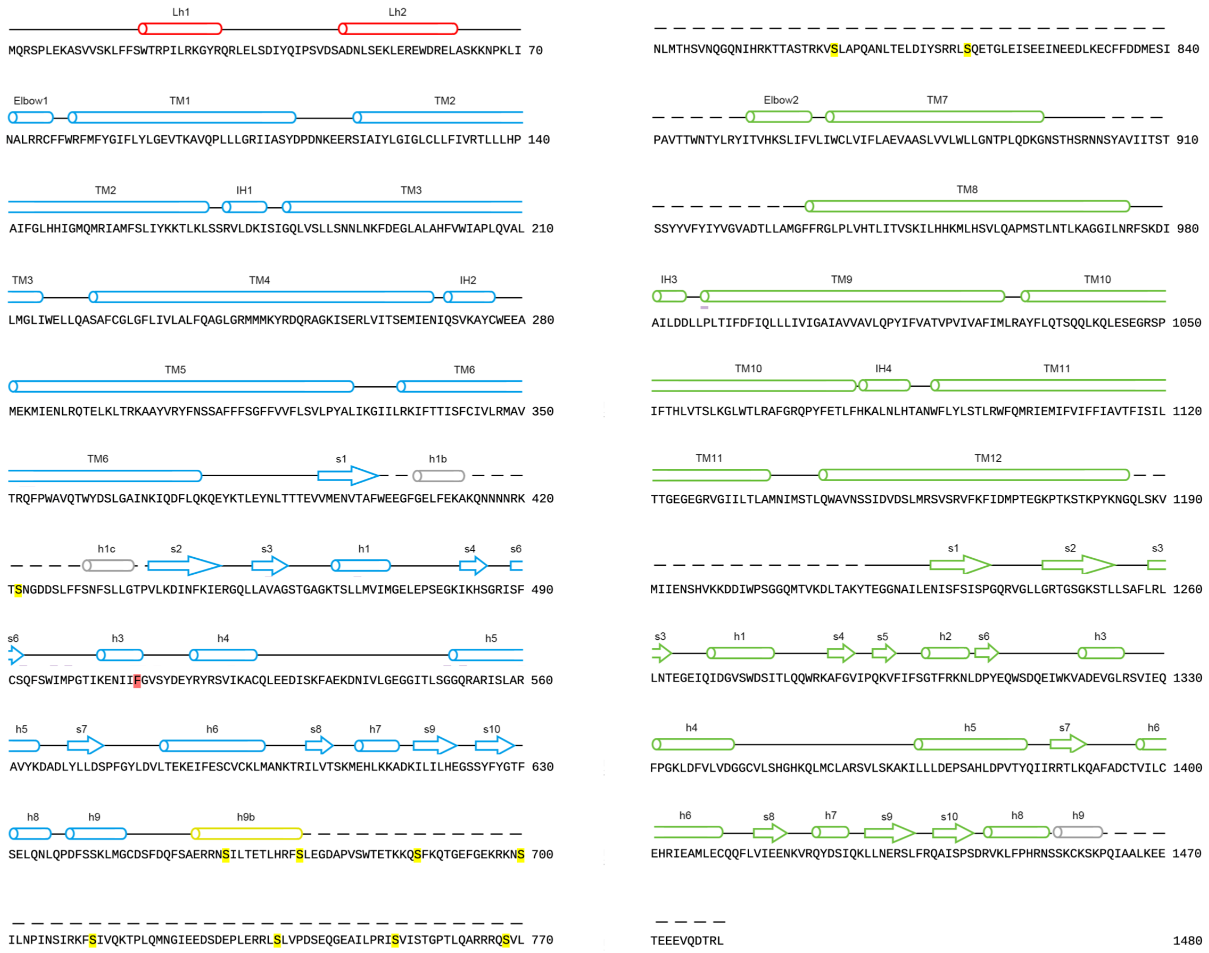

Figure S1. The human CFTR sequence with secondary structure indicated. The serines phosphorylated by PKA (ten in the R domain and one in NBD1) are highlighted in yellow. The position of the most common mutation, the ΔF508 site, is highlighted in red.

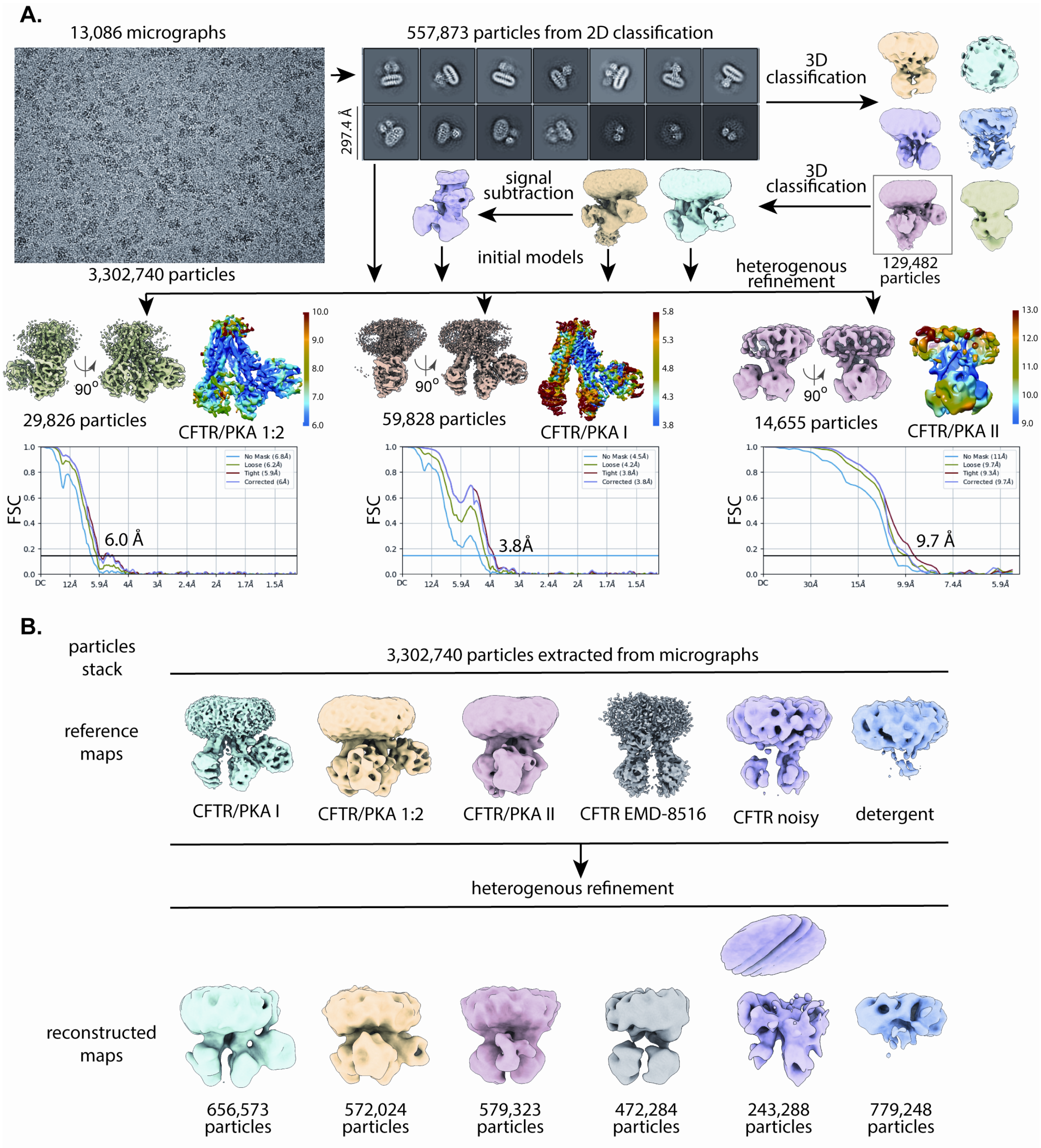

Figure S2. Cryo-EM reconstructions of PKA-C in complex with dephosphorylated CFTR

(A) Summary of image processing procedures and estimated resolution of each structure based on Fourier shell correlation (FSC) curves.

(B) Heterogeneous refinement of the entire dataset. Six structures were used as references for classification.

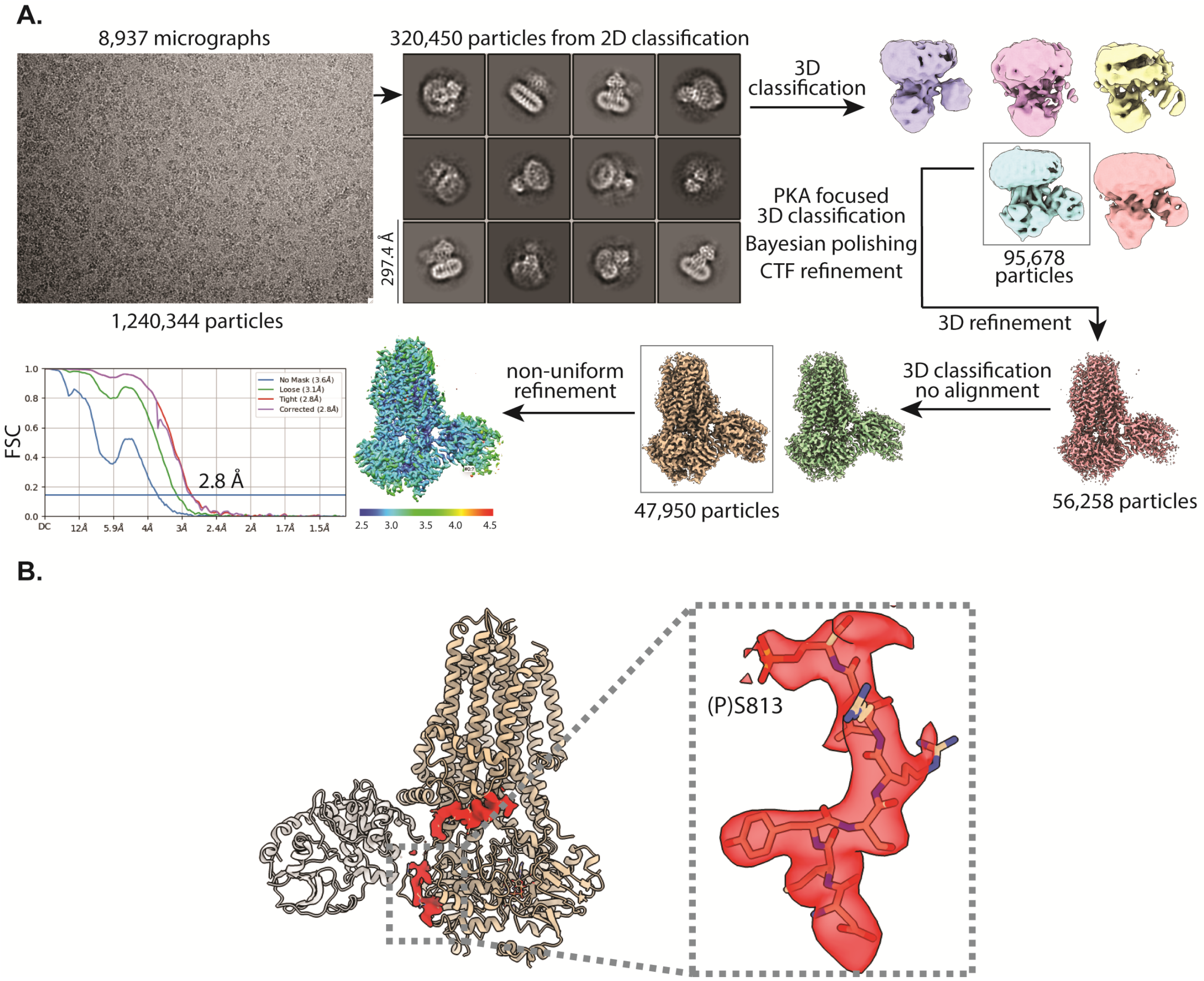

Figure S3. Cryo-EM reconstructions of PKA-C in complex with phosphorylated CFTR

(A) Image processing procedures and estimated resolution of PKA-C in complex with fully-phosphorylated, ATP-bound CFTR (E1371Q).
(B) Density of residues 806-814 and 820-833 of the R domain.

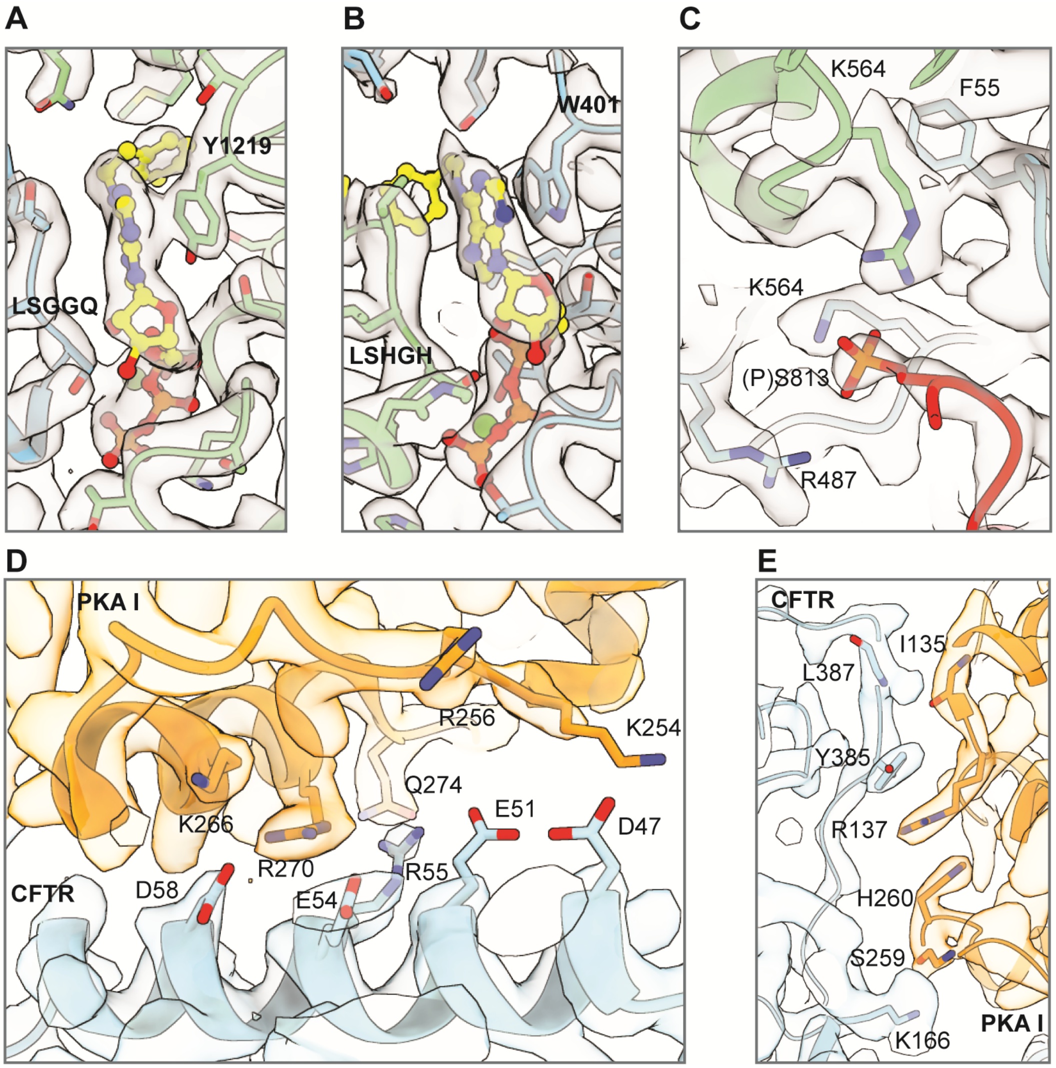

Figure S4. Zoomed-in view of the cryo-EM density of PKA-C in complex with phosphorylated CFTR

(A)(B) The two ATP-binding sites.
(C) The S813 phosphorylation site.
(D)(E) The CFTR/PKA-C interfaces.

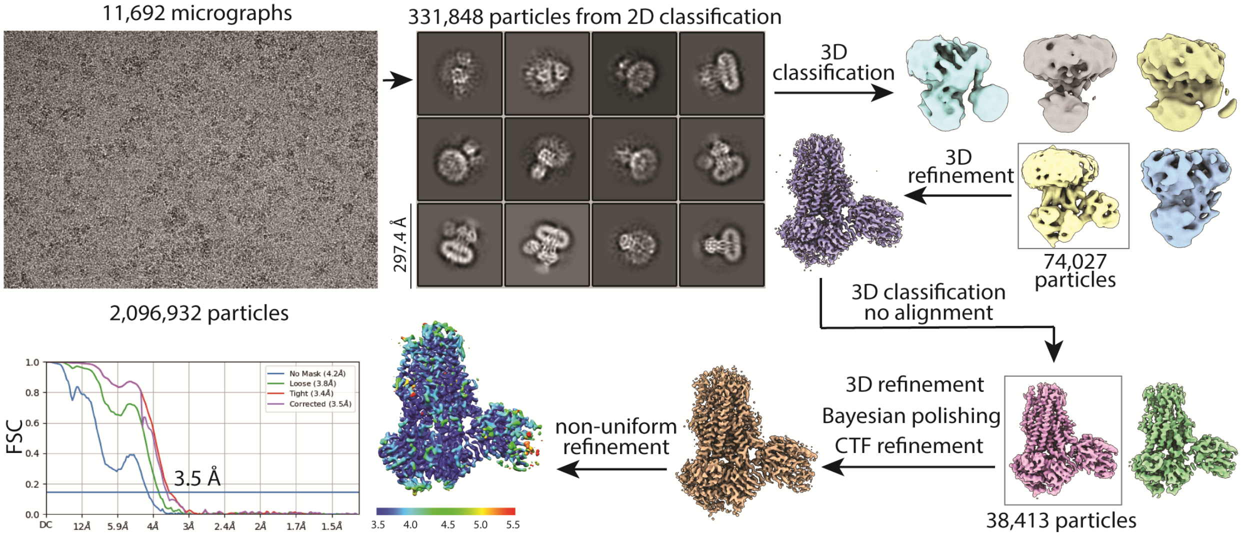

Figure S5. Cryo-EM reconstructions of PKA-C in complex with dephosphorylated CFTR, in the presence of P-ATP. Image processing procedures and estimated resolution of PKA-C in complex with dephosphorylated, ATP-bound CFTR (E1371Q) in the presence of P-ATP + AMPPNP.

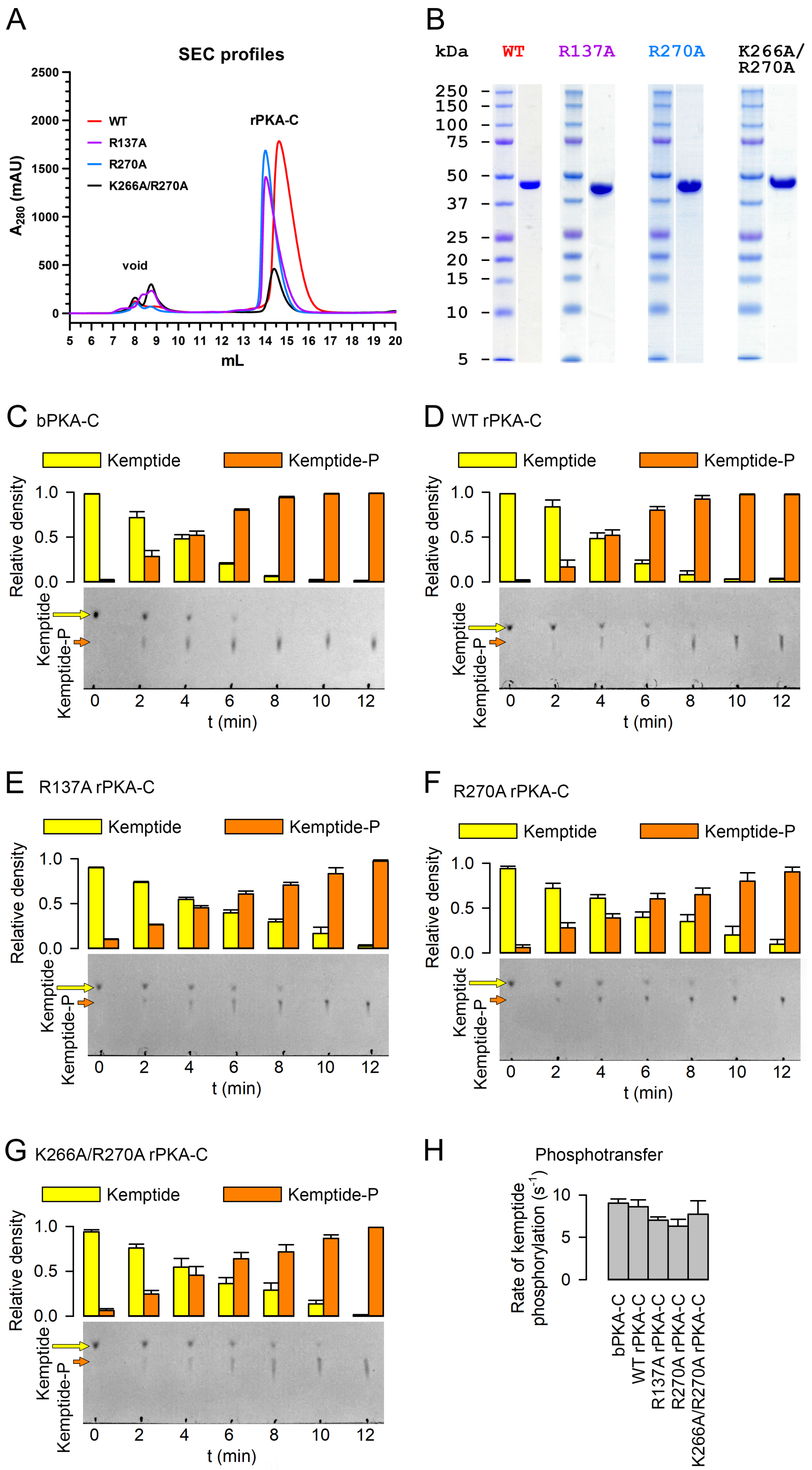
A
SEC profiles
A280 (mAU)
mL
WT
R137A
R270A
K266A/R270A
rPKA-C
void
B
kDa
WT
R137A
R270A
K266A/R270A
C bPKA-C
D WT rPKA-C
E R137A rPKA-C
F R270A rPKA-C
G K266A/R270A rPKA-C
Kemptide
Kemptide-P
Relative density
t (min)
H
Phosphotransfer
Rate of kemptide phosphorylation (s-1)
bPKA-C
WT rPKA-C
R137A rPKA-C
R270A rPKA-C
K266A/R270A rPKA-C

Figure S6. Purification of recombinant PKA-C (rPKA-C) constructs and rates of kemptide phosphorylation.

(A) Size-exclusion chromatography (SEC; Superdex 200 10/300 GL, GE Healthcare Hungary) elution profiles, detected as absorbance at 280 nm, of WT (*red*), R137A (*violet*), R270A (*blue*), and K266/R270A (*black*) rPKA-C.

(B) Coomassie-stained SDS PAGE gels of the final pooled fractions from (A). Molecular weights for marker ladder bands (Precision, Bio-Rad) are labeled in kDa.

(C)-(G); Time courses of kemptide phosphorylation resolved on TLC sheets (*Bottom*) and densitometric analysis (*Top*) for (C) bPKA-C, (D) WT rPKA-C, (E) R137A rPKA-C, (F) R270A rPKA-C, and (G) K266A/R270A rPKA-C. In each case 5 nM enzyme was incubated with 20 μM TAMRA-kemptide + 200 μM MgATP for the indicated amounts of time at room temperature, and 2 μl aliquots were spotted on the TLC sheets.

(H) Calculated k_{cat} (s^{-1}) for the five PKA-C proteins in (C)-(G). Data in (H) represent mean±SEM, n=3.

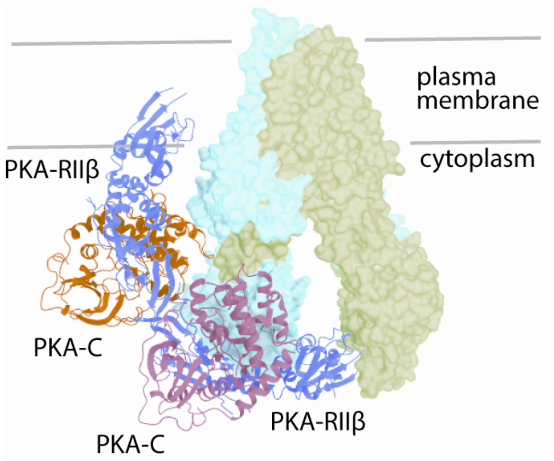

Figure S7. Alignment of PKA holoenzyme structure with CFTR-PKA $_{I}$ complex. Structure of dephosphorylated CFTR-PKA$_{I}$ complex aligned with the structure of the PKA $(C\alpha)_2(R\beta II)_2$ holoenzyme (PDBID: 3TNP). CFTR is shown as surface, PKA$_{I}$ as orange ribbon. For the PKA holoenzyme (ribbon) the R subunits are shown in blue, the C subunits in purple. One C subunit of the holoenzyme was aligned with PKA$_{I}$.

Table S1: Summary of data collection, processing, and model refinement

Dataset	De-phosporylated CFTR/PKA-C			Phosphorylated E1371QCFTR/ PKA-C	De-phosphorylated E1371QCFTR/ PKA-C
Structure	CFTR-PKA 1:2	CFTR-PKA I	CFTR-PKA II		
Cryo-EM Maps					
Microscope	Titan Krios G2	Titan Krios G2	Titan Krios G2	Titan Krios G2	Titan Krios G2
Camera	Gatan K3	Gatan K3	Gatan K3	Gatan K3	Gatan K3
Collection software	Serial EM	Serial EM	Serial EM	Serial EM	Serial EM
Magnification	105,000	105,000	105,000	105,000	105,000
Voltage (kV)	300	300	300	300	300
Total electron exposure ($e^-/Å^2$)	52	52	52	52.5	68.6
Exposure rate (e^-/pix/s)	19.8	19.8	19.8	20	20.9
Total frames	40	40	40	40	50
Defocus range (μm)	0.8-1.8	0.8-1.8	0.8-1.8	0.8-1.8	0.8-1.8
Pixel size (Å)	0.676	0.676	0.676	0.676	0.676
Micrographs collected	13,086	13,086	13,086	8,937	11,692
Initial particle images (no.)	3,302,740	3,302,740	3,302,740	1,240,344	2,096,932
Final particle images (no.)	29,826	59,828	14,655	47,950	38,413
Symmetry	C1	C1	C1	C1	C1
Map resolution masked (Å)	6	3.8	9.7	2.8	3.5
FSC threshold	0.143	0.143	0.143	0.143	0.143
Structural Models					
Initial model used (PDB and Alphafold codes)	5UAK AF-P00517-F1	5UAK AF-P00517-F1	5UAK AF-P00517-F1	6O1V AF-P00517-F1	6O1V AF-P00517-F1
Refinement Package	Phenix	Phenix	Phenix	Phenix	Phenix
Model resolution (Å)	6.3	4.1	13	2.9	3.7
FSC threshold	0.5	0.5	0.5	0.5	0.5
Non-hydrogen atoms	9039	9843	7331	12241	12026
Protein residues	1804	1464	1465	1511	1491
Ligands	3	3	0	12	7
CC masked	0.82	0.78	0.66	0.87	0.86
B factors ($Å^2$) mean					
Protein	74.4	120.3	758.9	52.3	43.6
Ligands	166.3	205.8	N/A	50	48
R.m.s. deviations					
Bond lengths (Å)	0.002	0.002	0.003	0.003	0.003
Bond angles (°)	0.447	0.62	0.59	0.58	0.69
Models Validation					
MolProbity score	1.1	1.8	1.7	1	1.5
Clashscore	1.5	7	7.1	1.7	3.8
Poor rotamers (%)	0	2	0	0.8	1.3
CaBLAM outliers	2	2	1.9	1.7	3
Ramachandran plot					
Favored (%)	96.5	97.1	95.2	97.9	97.2
Allowed (%)	3.5	2.8	4.7	2.1	3.8
Disallowed (%)	0	0.1	0.1	0	0
